# Opportunistic Strains of *Saccharomyces cerevisiae*: A Potential Risk Sold in Food Products

**DOI:** 10.3389/fmicb.2015.01522

**Published:** 2016-01-08

**Authors:** Roberto Pérez-Torrado, Amparo Querol

**Affiliations:** Food Biotechnology Department, Instituto de Agroquímica y Tecnología de los Alimentos – Consejo Superior de Investigaciones CientíficasValencia, Spain

**Keywords:** yeast, *S. cerevisiae*, food, opportunistic, infection

## Abstract

In recent decades, fungal infections have emerged as an important health problem associated with more people who present deficiencies in the immune system, such as HIV or transplanted patients. *Saccharomyces cerevisiae* is one of the emerging fungal pathogens with a unique characteristic: its presence in many food products. *S. cerevisiae* has an impeccably good food safety record compared to other microorganisms like virus, bacteria and some filamentous fungi. However, humans unknowingly and inadvertently ingest large viable populations of *S. cerevisiae* (home-brewed beer or dietary supplements that contain yeast). In the last few years, researchers have studied the nature of *S. cerevisiae* strains and the molecular mechanisms related to infections. Here we review the last advance made in this emerging pathogen and we discuss the implication of using this species in food products.

## Introduction

Fungal infections are an extremely important health problem. According to numerous studies, *Candida albicans* and other *Candida* species are the most remarkable pathogenic fungi which cause some 7000-28000 nosocomial infections annually (Pfaller and Diekema, 2007). The general characteristic of fungal infection is that it is produced as a result of reduced immunity. Most fungal pathogens are classified as opportunistic. This concept implies that under normal conditions, these organisms are not capable of producing infection but, when host defenses are weakened, there is room for them to prosper and to generate a health problem. Another general characteristic of fungal infections is that they are frequently moderated and localized. However, fungal pathogens are able to produce fungal disease, systemic infection, and even death in the worst scenarios.

In the last century, fungal infection cases have dramatically increased, especially in developed countries. One work has shown that the number of cases of sepsis produced by fungal organisms in the USA has increased by 207% between 1979 and 2000 (Martin et al., 2003). This phenomenon is associated with the appearance of medical techniques, such as the use of broad-spectrum antibiotics, the use of intravenous catheters, how intensive care units are organized, increased number of organ transplants, or the development of cytotoxic chemotherapies. On top of that, pandemics like HIV/AIDS have exponentially increased the number of patients with impaired immunity. In fact, fungal disease was extremely rare before all these changes occurred.

A paradigm of an emerging fungal organism is the yeast *Saccharomyces cerevisiae*. This species can be found naturally in many niches in the environment, but is most commonly known for its role as “baker’s yeast” in either traditional or industrial fermentative production of bread, beer or wine. It has also been used as an agent to treat antibiotic-related diarrhea and as a nutritional supplement, when it is commercialized as *S. boulardii*. Classically, *S. cerevisiae* has been considered a safe non pathogenic organism. However in the last two decades, the number of cases of diagnosed infections has increased, probably as a result of the increased numbers of immunocompromised patients, but also due to advances made in diagnostic methodologies in hospitals, including genetic identification by molecular techniques. *S. cerevisiae* has been related to a wide variety of infections, which range from vaginitis in healthy patients and cutaneous infections, to systemic bloodstream infections and infections of essential organs in immunocompromised and critically ill patients (Enache-Angoulvant and Hennequin, 2005; Muñoz et al., 2005; de Llanos et al., 2011). Infected patients tend to be elderly people, premature children or patients suffering from immunosuppression due to HIV/AIDS, treatment with immunosuppressive agents, or other conditions associated with a deficient immune response. Furthermore, severe infections with *S. cerevisiae* have been occasionally reported in patients with no obvious predisposing factors (Jensen and Smith, 1976; Smith et al., 2002). All these data have changed the status of *S. cerevisiae*, which is now considered an emerging opportunistic pathogen (Herbrecht and Nivoix, 2005; de Llanos et al., 2006).

## *S. cerevisiae* Population Diversity: Opportunistic Strains

The species *S. cerevisiae* is very heterogeneous and contains strains with specific abilities like sherry wine strains, *S. boulardii* or baker strains. Before the development of high throughput sequencing techniques, the population structure of *S. cerevisiae* was not very clear. Now we know that it is structured into several genetically pure subpopulations and many mosaic strains that contain gene alleles of different subpopulations (Liti et al., 2009). In the last decade, yeast scientists have attempted to determine if the strains isolated from infected patients form a specific *S. cerevisiae* subpopulation with any special characteristic. de Llanos et al. (2004) used molecular markers as mt DNA restriction patterns and showed that clinical strains were present in several genetically differentiated groups of strains. In contrast, Carreto et al. (2008) used comparative genome hybridization on array (aCGH), and suggested that clinical strains could be a genetically homogenous subpopulation. Later, Wei et al. (2007) sequenced the genome of *S. cerevisiae* strain YJM789 derived from a yeast isolated from the lung of an AIDS patient with pneumonia. Liti et al. (2009) sequenced 36 new strains that contained six clinical isolates. Strope et al. (2015) sequenced 93 strains from multiple geographic and environmental origins, including several clinical strains. Finally, after the sequencing of the whole genome of several clinical strains, it turns out that they are not a genetically homogenous group of strains, but are all mosaic strains with relatively heterogeneous genetic content (Liti et al., 2009; Strope et al., 2015).

Several studies have analyzed the potential virulence of this yeast species *in vitro* (McCusker et al., 1994; de Llanos et al., 2006) and *in vivo* (McCullough et al., 1998; de Llanos et al., 2011), and have suggested that some strains have the potential to cause disease regardless of their clinical or non clinical isolation origin. Many strains of *S. cerevisiae*, which have been isolated in clinical settings, present very low levels of virulence in mice infection models, while other strains, such as strain D14 isolated from a dietetic supplement, have shown a relative high level of virulence in different infection models (Llopis et al., 2012, 2014; Pérez-Torrado et al., 2015). Thus we propose that the term “opportunistic strain” is used more accurately and usefully to categorize these strains rather than “clinical strains” since not all clinical strains cause infections. Even more importantly, some non clinical strains can cause infections. We define opportunistic *S. cerevisiae* strains as those strains which show physiologic characteristics of yeast pathogens, such as growth, at 37 °C (McCusker et al., 1994; de Llanos et al., 2006), but unlike most other strains, can also cause infections and kill mice (de Llanos et al., 2011). These strains also survive better in human blood infection models than other strains, which may enable them to disseminate across the body and reach organs under adequate propagation conditions and in certain circumstances (Lin et al., 2012). Increased blood survival can be a key feature to distinguish these opportunistic yeasts strains from others.

## *S. cerevisiae* Infection Mechanisms

The infection mechanism of *C. albicans*, a fungal pathogen that is phylogenetically close to *S. cerevisiae*, is based on a first step of adhesion to human surface tissues and uses a family of proteins called adhesins. *C. albicans* is also able to penetrate epithelial or endothelial barriers via active mechanisms (Dalle et al., 2010). A second important aspect of infection mechanisms is resistance to the stressful situations generated after an encounter with cells of the immune system. In contrast, *S. cerevisiae* lacks homologous genes for the well-known *C. glabrata* or *C. albicans* adhesins and shows low adhesion levels to human tissues compared to both *Candida* species (Pérez-Torrado et al., 2012). It has been suggested that *S. cerevisiae* can only perform opportunistic or passive crossings when epithelial barrier integrity is previously compromised (Pérez-Torrado et al., 2012).

**FIGURE 1:**
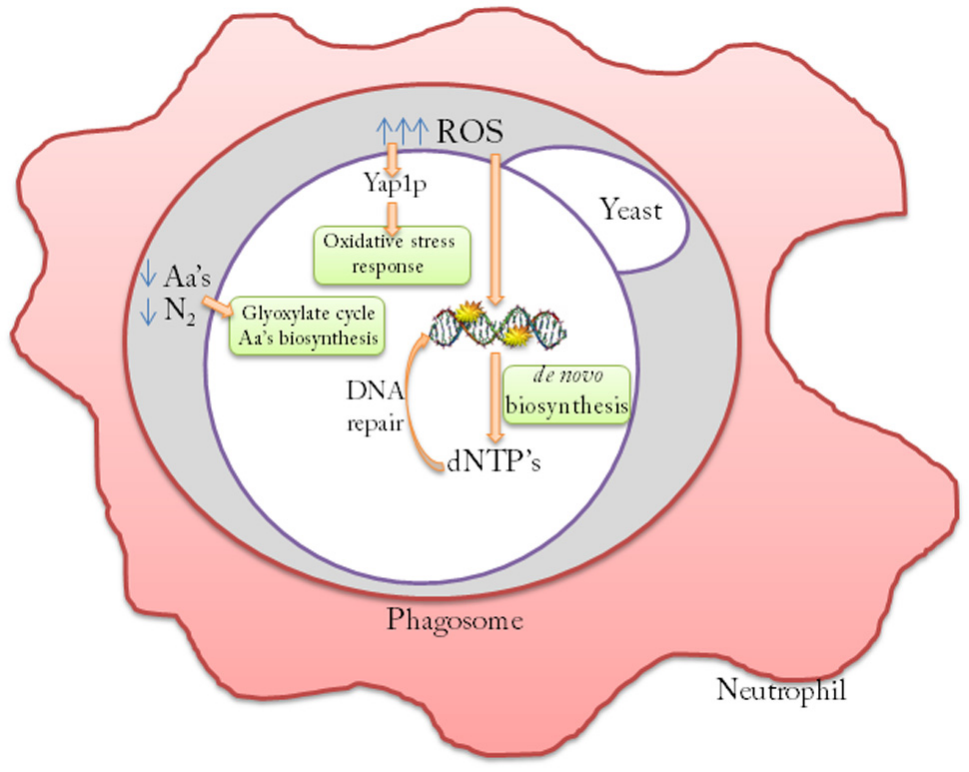
**Molecular mechanisms of persistence of *Saccharomyces cerevisiae* cells after neutrophil engulfment.** Opportunistic *S. cerevisiae* strains redirect the low levels of the nitrogen sources inside the phagosome to increase amino acids (aa’s) biosynthesis. These strains also increase the oxidative stress response by activating the Yap1p transcription factor regulon to counteract the lethal effects of reactive oxygen species (ROS) generated by the macrophage. *De novo* dNTP biosynthesis is activated to allow DNA repair machinery to counteract ROS-induced mutations.

However, previous studies by our group (Llopis et al., 2012) have demonstrated that opportunistic *S. cerevisiae* strains show a specific transcription pattern after human blood infection, which reflects a specific oxidative stress response, increased amino acid biosynthesis and a DNA damage repair response (**Figure 1**).

Yeast pathogens are adapted to resist human defenses and one of the main responses of the immune system is microbe engulfment and oxidative burst. Neutrophils, macrophages and other cells with phagocytic capacity generate potent reactive oxygen and nitrogen species (ROS and RNS), which can be lethal to most fungal pathogens by causing damage to DNA, proteins and lipids (Bogdan et al., 2000). Most fungal pathogens display resistance to the reactive oxygen and nitrogen species used by human cells to counteract infection (Brown et al., 2009). Fungal resistance to ROS offers protection from oxidative host defenses and is undoubtedly an advantageous pathobiological property (Lushchak et al., 2010; Rodrigues-Pousada et al., 2010). It has been described that pathogens such as *C. albicans* or *C. neoformans* have the potential to resist, among other suboptimal conditions, oxidative stress produced by the ROS generated in the phagosome (Brown et al., 2007, 2014). Indeed the thioredoxin system of *C. albicans* and *S. cerevisiae* has been shown to be expressed during growth in human blood or mucosal tissue (Fradin et al., 2003, 2005, Zakikhany et al., 2007; Llopis et al., 2012), which indicates that the ability to respond to oxidative stress might be crucial in early stages of systemic infections. *TRX1* (thioredoxin 1) is also necessary to survive the oxidative environment of macrophages in *C. neoformans*, and is important for the virulence of this fungal pathogen (Missall and Lodge, 2005). The *TSA2* (thioredoxin peroxidase 2) and *GPX2* (glutathione peroxidase 2) genes have been shown to be induced in *S. cerevisiae* strains when exposed to neutrophils (Rubin-Bejerano et al., 2003), and a clear antioxidant response has been observed. Fradin et al. (2005) demonstrated that neutrophils play a key role in bloodstream infections with *C. albicans*. This observation is in line with the high susceptibility shown by neutropenic patients (deficient in these immune cells) to disseminated candidiasis (Bodey et al., 1992; Wright and Wenzel, 1997). In *S. cerevisiae*, the main role of proper oxidative stress response virulence has been suggested since a virulent strain mutant in transcription factor Yap1p, the main transcription factor involved in oxidative stress response that is unable to grow under oxidative stress conditions, presented low survival levels in human blood compared with the wild type or the *YAP1* reconstituted strain (Llopis et al., 2012). Diezmann and Dietrich (2011) compared hundreds of clinical isolates and showed that they were more resistant to oxidative stress after they verified the central role of oxidative stress resistance in *S. cerevisiae* virulence.

After exposure to human neutrophils or cultured macrophages, *C. albicans* cells up-regulate amino acid biosynthetic genes (Rubin-Bejerano et al., 2003; Fradin et al., 2005). Rubin-Bejerano et al. (2003) observed induction for these pathways after *C. albicans* and *S. cerevisiae* cells were ingested by neutrophils. This suggests that the microenvironment in the phagosome inside the neutrophil is deficient in amino acids, and generates a rapid response from yeast strains. The methionine and arginine biosynthetic genes are induced when *S. cerevisiae* is phagocytized by the murine macrophage-like cell line (Lorenz and Fink, 2001). Kingsbury et al. (2006) revealed the relevance of amino acid biosynthesis for yeast survival in a murine host and suggested that yeast can use a variety of nitrogen sources under these conditions. The glyoxylate cycle is also induced upon phagocytes ingestion of the bacterium *Mycobacterium tuberculosis* (McKinney et al., 2000), and other fungi such as *C. neoformans* (Rude et al., 2002), *Leptosphaeria maculans* (Idnurm and Howlett, 2002), *C. albicans* (Lorenz and Fink, 2001), and *S. cerevisiae* (Lorenz and Fink, 2001). This change in metabolism is a response to the glucose-poor environment of the macrophage, and contributes to the virulence of some pathogens. The *ICL1* gene, which encodes for isocitrate lyase, one of the principal enzymes of the glyoxylate cycle, has been recently shown to be substantially induced upon exposure to macrophages *in vitro* in both *S. cerevisiae* and *C. albicans* (Lorenz and Fink, 2001; Lorenz et al., 2004). All these data suggest that the *ICL1* gene may also play a general role in *S. cerevisiae* in human infections.

A common mechanism described for human microbial pathogens is to increase dNTP pools in order to repair the DNA damage caused by the oxidative burst of phagocytes. The importance of a *de novo* nucleotide biosynthetic pathway for phagocyte survival has been demonstrated for bacterial pathogens such as *Salmonella enterica* (Panosa et al., 2010), *Bacteroides fragilis* (Smalley et al., 2002), *Pseudomonas aeruginosa* (Sjöberg and Torrents, 2011), *Staphylococcus aureus* (Kirdis et al., 2007), *Streptococcus pyogenes* (Le Breton et al., 2013), *Bacillus anthracis*, and *Escherichia coli* (Samant et al., 2008). The relevance of *de novo* nucleotide biosynthetic pathways has also been implicated for fungal pathogens like *Cryptococcus neoformans* (Morrow et al., 2012) or *C. albicans* (Donovan et al., 2001; Jiang et al., 2010), and nucleotide biosynthetic pathways have been discussed as a target for antifungal compounds (Rodriguez-Suarez et al., 2007). In a recent study, we observed that *S. cerevisiae* opportunistic strains, like other human pathogens, have the enhanced ability to produce dNTPs, the substrates used by DNA repair machineries (Pérez-Torrado et al., 2015). The importance of this pathway for the virulence of *S. cerevisiae* has been confirmed by experimental infections conducted in immunodeficient murine models using a Δ*gua1* mutant, which is a key enzyme for *de novo* dNTP biosynthesis. Additions of exogenous guanine and the use of mutants in the DNA damage checkpoint, which activates dNTP biosynthesis in yeast cells, affects the survival of yeast cells in *ex vivo* blood infections (Pérez-Torrado et al., 2015). The nitrogen source preferentially used by yeasts in phagosomes to increase this pathway is still unknown.

## Conclusion and Perspectives

In developed countries, consumers have been driven to take a more critical attitude about what they eat and drink as a requirement of modern life. Food microbiologists are facing the huge challenge of regarding food freshness that is implicit in consumer demand for more natural products. Additive-free safer food with less severe processing that has a satisfactory shelf life and is easy to prepare is required given the greater awareness of nutrition and health. These changes in consumer preferences, which modify food processes, may have important consequences and could affect both food quality and safety. Yeasts, especially *S. cerevisiae*, have an impeccably good food safety record compared to other microorganisms like virus, bacteria and some filamentous fungi. However, humans unknowingly and inadvertently ingest large, viable populations of *S. cerevisiae* without them having adverse impacts on their health (e.g., yeasts in home-brewed beer, beers enriched by yeast or dietary supplements that contain yeast are very common today). Nevertheless, having an open mind about, and conducting vigilance of, yeasts and food-borne diseases are required. Compared with other microbial groups, yeasts are not seen as aggressive pathogens, but they are capable of causing human disease in opportunistic circumstances (Puig-Asensio et al., 2014). As we summarize in this paper, yeast researchers have made progress in understanding the nature and virulence mechanism in *S. cerevisiae* strains. In the future, more attention has to be paid to industrial practices that are more prone to generate opportunistic *S. cerevisiae* strains.

## Conflict of Interest Statement

The authors declare that the research was conducted in the absence of any commercial or financial relationships that could be construed as a potential conflict of interest.
